# Acknowledgement to Reviewers of *Genes* in 2019

**DOI:** 10.3390/genes11010059

**Published:** 2020-01-04

**Authors:** Genes Editorial Office

**Affiliations:** MDPI, St. Alban-Anlage 66, 4052 Basel, Switzerland

Rigorous peer-review is the corner-stone of high-quality academic publishing. The editorial team greatly appreciates the reviewers who contributed their knowledge and expertise to the journal’s editorial process over the past 12 months. In 2019, a total of 1057 papers were published in the journal, with a median time to first decision of 22.46 days and a median time to publication of 42 days. The editors would like to express their sincere gratitude to the following reviewers for their cooperation and dedication in 2019:
Aakrosh RatanKathrin BlagecAaron StevensKathrin MaedlerAbhijeet A. BakreKathrin MarheinekeAbhijit KaleKatja StangeAbhinandan PatilKatsuharu SaitoAbhinav KaushikKausik DattaAbul UsmaniKay LucekAchim C. SchnauferKazue NagasawaAdam BewickKazufumi MochizukiAdam CawleyKazuko Koshiba-TakeuchiAdam CribbsKazuo NakashimaAdam EwingKe XuAdam J. KriegKedryn. BaskinAdam StewartKelly A. MeiklejohnAdebowale OgunjirinKelly ElkinsAdolfo SaiardiKelly M. BakulskiAdrian DockeryKen W.Y. ChoAdrian MorenoKen-ichiro MinatoAdrian Ochoa-LeyvaKenji FukunagaAdrian VolceanovKenji HikosakaAdriana GarayKenneth StedmanAdvaitha MadireddyKensaku SakamotoAgata Kryczyk-PoprawaKensuke FurukawaAgda Karina B. EterovicKeren MacholAglaia PappaKerstin Lindblad-TohAgnes SzepesiKeshav Raj PaudelÁgnes TantosKevin BoothAgnieszka FiszerKevin HardwickAgnieszka Marasek-CiolakowskaKevin Van BortleAhmad OmarKhulan BatbayarAhmed ArslanKhurshid AhmadAi KitazumiKi-Hong JungAida Martinez-SanchezKim Tae-JinAiden Eliot ShearerKimberly GreerAimy SebastianKimberly M. WebbAkhtar AliKin Fai AuAkihiro UedaKirsten WalenAkiko KozakiKirsty ShortAkiko SugaKiyoshi KikuchiAkimitsu TakagiKiyoshi SaigusaAkira MutoKiyotaka NagakiAkira SuzukiKlaus StegerAkira YamashitaKlaus WimmersAkram AlyassKoichi OgamiAlain PerretKoji SatoALAN B. BennettKonstantin PopadinAlan ChambersKotaro KitayaAlan EalyKouichi KawamuraAlan WatsonKranthi VaralaAlbert De La ChapelleKrishan RaiAlejandro Facundo Barrientos-PriegoKristina DøssingAlejandro MoralesKristine FreudeAleksandra KoracKruttika BhatAleksey KomissarovKui ZhaiAles EichmeierKULDEEP GUPTAAleš PečinkaKurt FagerstedtAlessandra FaveroKurt WeisingAlessandro De LucaKurt Werner SchmidAlessandro GrapputoKwang Poo ChangAlessio BechiniKwon-Kyoo KangAlessio MengoniKwon-Yul RyuAlessio MetereKyle TretinaAlessio PapiniKylie CairnsAlessio SquassinaKyu-Sang LimAlessio ValentiniLadislav BocakAlexander A. MakhrovLaia Rodriguez-RevengaAlexander AwgulewitschLan XiongAlexander BrobeilLarry CroftAlexander GraphodatskyLars H. WegnerAlexander KrivoruchkoLaura Alejandra De La RosaAlexander L. GreningerLaura BurrackAlexander LorenzLaura Lindsey-BoltzAlexander PeltzerLauréline RogerAlexander SwidsinskiLauren M. OsborneAlexandre MartinièreLauren WhiteAlexei N. FedorovLawrence J. JenningsAlexey I. MakuninLee Bulla JrAlexey I. MakuninLee WongAlexey KolodkinLeena Hilakivi-ClarkeAlexey MoskalevLei ShiAlexis JanosikLei WangAlfonso SenatoreLeigh Anne ClarkAlfonso UrbanucciLenka SkálováAlfred Edward SzmidtLenka StixovaAli AliLeonor PereiraAlicja PacholewskaLeos G. KralAline TalhoukLeslie TurnerAlison L. GouldLev PorokhovnikAlistair J. StandishLewis FreyAllison BragerLi FanAlok SharmaLiang NiuAmalio TelentiLily MomperAmanda AmodeoLin DingAmanda LarracuenteLindsay FryAmarda ShehuLindsey Van HauteAmelia CasamassimiLingling AnAmit R. ReddiLionel VerdoucqAmit SinhaLiqing ChenAmnon LersLisa M. NilssonAmol C. ShettyLisa TimmonsAmy L. StarkLise PingaultAmy V WhippleLiu FilipeAn GorisLivia DonaireAna ButrónLivia GaravelliAna Clara AprotosoaieLivia UlicnaAna ConesaLleretny Rodríguez AlvarezAna CrnkovicLloyd W. RuddockAna GradissimoLolitika MandalAna I. SoteloLong GaoAna MuñozLongzhi TanAna Paula SantosLoredana CiarmielloAna Silvia A. M. T. MouraLoredana MoroAnastasios D. TsaousisLorraine SymingtonAnders Palmstrøm JørgensenLouise NewnhamAndi M. WilsonLourdes Ruiz DesviatAndras PerlLuca BelloAndre Van WijnenLuca BragliaAndrea BagiLucia BongiorniAndrea Clavijo-McCormickLucia Vieira HoffmannAndrea CorsinottiLucie LáníkováAndrea LuchettiLucy OsborneAndrea TrevisanLudovic DutoitAndreaja Čanžek MajheničLuis BotoAndreas FischerLuis Diaz-GarciaAndreas KreutzerLuis Monteagudo IbañezAndreas R. HuberLuisa Garcia-OliveiraAndrei Cristian GradinaruLuisa GarofaloAndrei DragomirLuisa PallaresAndrei HerdeanLukáš KratochvílAndrej ŠušorLukasz DziewitAndres J. CortesŁukasz OpalińskiAndrés Posso-TerranovaLukasz SydlowskiAndreu Comas-GarciaLuke C. PillingAndrew BeckenbachM. Carmen LimónAndrew F. RowleyM. Luisa HernándezAndrew FooteM. StaceyAndrew JohnstonMaarten KoornneefAndrew MitchellMacarena Cabrera-SerranoAndrew P HutchinsMaciej TarnowskiAndrew PatersonMaciej WnukAndrew SchurkoMackenzie GaveryAndrew SeeberMad BallAndrew ShawMadhav BhattaAndrew SweetMadhusudana Reddy JangaAndrew TosoliniMadhusudhana JangaAndrew WallerMadison S. PowellAndrey A. YurchenkoMagali OlivierAndrey ZamyatninMagdalena PaplinskaAndrzej PawlikMagdalena SimlatAndy D. HerringMagriet A Van Der NestAneta BalcerczykMahantesh KurjogiAnfu HouMahesh NeupaneAngela C. HirbeMahmoud NaguibAngela G. FleischmanMaija CastrenAngela Roberta Lo PieroMaja GregićAngela RoggeroMaja LeitgebAngeliki AsimakiMalene LindholmAnindita DasMałgorzata Anna MarćAnja Katrin BielinskyMałgorzata Karbownik-LewińskaAnn HorsburghMalgorzata MaciukiewiczAnn KirchmaierMali Salmon-DivonAnna Bilska-KosMaliheh MehrshadAnna CieślińskaMalin BombergAnna Di CosmoManel Vera RodríguezAnna GałązkaManjul SinghAnna Junkiert-CzarneckaManuela SalvucciAnna KashinaMarc GartenbergAnna La TorreMarc GrailleAnna M. TracewskaMarc ServantAnna MediciMarçal GallemíAnna OtlewskaMarc-André SirardAnna TangMarcel MéchaliAnna Tracewska-SiemiątkowskaMarcel TijstermanAnna Y. AksenovaMarcella CervantesAnnabel WhibleyMarcello MezzasalmaAnne C. EischeidMarcelo Ricardo VicariAnne ChenuilMarcio DornAnne HenstraMarco ArculeoAnne KupczokMarco CastoriAnne OsbournMarco CicciùAnne PiantadosiMarco MascittiAnne-Kathrin ClassenMarco Matucci-CerinicAnne-Sophie Vercoutter-EdouartMarco SazziniAnnette Payne Marcos YanniccariAnnette SchenckMarcus OlatoyeAnnie RobicMarek SkrzypskiAnsgar GruberMarek ŚwitońskiAnte IvankovicMargarita MeerAnthony FedeleMargherita CerroneAnthony MusolfMargie ClapperAnthony VandersteenMaria Chiara FabbriAntoinette Van Der KuylMaria Adelaide CaligoAnton JettenMaria Anna SmolleAnton NikolaevMaría Antonia Alvarez FernandezAnton SonnenbergMaría Auxiliadora Dea-AyuelaAntonia ChroniMaria BatoolAntonio BlancoMaría Belén PascualAntonio CarapelliMaria Chiara AnaniaAntonio FerranteMaria De La O Leyva PérezAntonio González-MartínMaria Doroteia CamposAntonio Humberto H. MinervinoMaría Esther EstebanAntonio José Piantino FerreiraMaria IbarsAntonio Juan PazosMaría Jesús HerreroAntonio Juárez-MaldonadoMaria Lucia Z DagliAntonio MarcoMaria NilssonAntonio MasMaria S MuntyanAntonio PercesepeMaria SharakhovaAntonio RivaMaria TrojanowskaAntonio SánchezMarian BeekmanAntonis GoulasMarian BresticAntony DoddMarian Price-CarterAnurag SinghMariano StornaiuoloAref Arzan ZarinMaribel Forero-CastroAreti StratiMarie UmberArie GeerlofMarie-hélène David-CordonnierAristotelis PapageorgiouMarieke VerleihArkadiusz OrzechowskiMariella Matilde Finetti-SialerArley CamargoMarija VujadinovicArmita ZarnegarMarika KaakinenArnab GhoshMarika VitaliArnaud PommierMariko Taniguchi-IkedaAroa Suarez-VegaMarilena BazzanoArtur AlvesMarina CaldaraArtur GurgulMarina KlemenčičArtur JarmolowskiMarina NaoumkinaArun KumarMarina R.S. FortesArun SeetharamMarina SoleAsha KumariMario De FeliceAshraf El-KereamyMarion PilletAssunta BertacciniMarissa SobolewskiAtlanta G. CookMarjorie JonesAtsushi OdaMark J. UlineAtsushi SakaiMark E. CorkinsAtul KakranaMark MaconochieAtul SrivastavaMark ParcellsAugusto OrlandiMark WidrlechnerAurelie GoyenvalleMarkus J. HerrgårdAuriel A. WilletteMarkus KuhlmannAurora Ruiz-HerreraMarkus NeuditschkoAvinash Chandra PandeyMarkus RuhsamAvinash ShanmugamMarla McPhersonAxel CournacMarta Berrocal-LoboAzusa InoueMarta Fernández MercadoAzza H. MohamedMárta MedveczBailin ZhaoMarta R. RomeroBalachandran ManavalanMarta TomaszkiewiczBalasubrahmanyam AddepalliMartha Carolina Chavarro FierroBam PaneruMarti AldeaBarbara BlasiMartin DruckerBarbara PatriziMARTIN KUHLWILMBarbara Schoepp-CothenetMartin PootBarbara Y WhitmanMartin VingronBartosz KozakMartin WabitschBashayer R. Al-MubarakMartin ZofallBegoña Renau-MorataMartina KroppBehnam AbashtMartina LariBeide FuMartina ZappaterraBen GreenebaumMartine DunnwaldBenedikt B. KauferMary C. MajBenjamin AnsaMarzia Di DonatoBenjamin L. SchulzMarzia Di FilippoBenjamin LangMarzia RossatoBenjamin RocheMasaharu HazawaBenoit ArveilerMasahiro KanedaBenoit ChénaisMasakatsu WATANABEBenoit GauthierMasako SuzukiBenoît Sessinou AssogbaMasashi TabuchiBerlin LondonoMasayoshi ShigyoBernard A. OkechMassimiliano CardinaleBernard CarrollMassimo BernardiBernard OkechMassimo BionazBernard RobaireMassimo Dal MonteBernardo BertoniMassimo La RosaBernd WemheuerMassimo LibraBernhard H.F. WeberMathias RheinBernie WoneMathilde CausseBertrand EardlyMatias MorinBertrand PainMatilde FernándezBeth SullivanMatteo BodiniBharani Krishna Mynampati ArunadithyaMatteo CeredaBharesh ChauhanMatteo MoriBhattacharya MadanMatthew AckermanBhupesh SinglaMatthew BakerBhushan ShresthaMatthew CollinBianca HabermannMatthew D BensonBin TianMatthew E. MeadBirte MöhlendickMatthew HarrisBjörn WallnerMatthew HillwigBlanca Vera-GargalloMatthew J. GreenwoldBlanche CapelMatthew R WillmannBo WangMatthew RogersBoas PuckerMatthew RutarBoby MathewMatthias BenoitBode OlukoluMatthias FutschikBohdan SchneiderMatthias Hardtke-WolenskiBon Ham YipMatthias SchaeferBonnie BaxterMattia ForcatoBoris EggerMattias CollinBraden MolhoekMaura DandriBreno FragomeniMaurice LingBrett D. KeiperMaurizio ChiurazziBrett T. ChiquetMaurizio MargaglioneBrian CalviMax KullbergBrian J. MorrisMax RobinsonBrian SayreMaxim MessererBrittney KeelMaximiliano AmenabarBruce A. RosaMaya RaghunandanBruno A C M SantosMayumi EndoBruno F. MeloMazahar MoinBruno Ramos-MolinaMegan KeniryBuket OnelMehanathan MuthamilarasanBurt AndersonMehdi MomenByung-Whi KongMehmet KayaCallum George DonnellyMehul VoraCamillo AutoreMelanie LegrandCandan HizelMelha MellataCara A. GriffithsMelis KucukogluCarina ScheyMelissa KeinathCarl Gordon JohnstonMelissa RichardCarl WhittingtonMelvin Y. RinconCarla CarducciMeng HanCarlo ContiniMeng How TanCarlo RiccardoMeng MaoCarlo VitiMeng-Chi YenCarlos E. AlvarezMeritxell JodarCarlos Enrique PradaMetin OzataCarlos FarinhaMichael A. TrakselisCarlos MenesesMichael ArabatzisCarlos VicientMichael BaumCarmen RizzoMichael C. GoldingCarmen Sato-BigbeeMichael CalcuttCaroline AustinMichael CampbellCaroline MarconMichael DarkCarrie J. FinnoMichael FieldCarrie Olson-ManningMichael G. CampanaCatalina Lopez-CorreaMichael GänzleCathelijne Van Der WoudenMichael HofreiterCatherine CoombsMichael HuberCatherine CullinghamMichael J. LeaverCatherine E. VrentasMichael JantschCatherine PeñaMichael KristensenCatrin MooreMichael LudwigCecilia BattistelliMichael P GustafsonCecilia QuirogaMichael PrimigCecilia TamborindeguyMichael T. EadonCesar De La FuenteMichael ThompsonCésar López-CamarilloMichael WeberCesar MartinsMichail RovatsosChandan PalMichal RurekChang-Bae KimMichal ZurovecChanon NgamsombatMichalis KaramouzisChantal Wicky CollaudMichel AudiffrenCharles BowmanMichel CharbonneauCharles E. SamuelMichel RavelonandroCharles PerrierMichele Crozatier-BordeChe-Kun James ShenMichele GiannattasioChen JiaoMichelino Di RosaChen ZhiqiangMichelle AllenCheng Ya-MingMichelle CareyChengdao LiMichelle GruninChenyang CaiMichelle ThomasCheryl Ingram-SmithMickael Le GacChiara DiquigiovanniMiguel E. Quiñones-MateuChien-Chung ChangMihai MiclăușChih-Jen YangMihai NitulescuChin Yan LimMikami KojiChloe ZubietaMike Dyall-SmithChong TengMikhail AlexeyevChongmei DongMikhail DivashukChris DraperMikhail Y. SyromyatnikovChris W. RogersMilena PetriccioneChristi GuerriniMin NiChristian Danve M. CastroverdeMin TuChristian MayerMin WuChristian P. SchaafMinoru TakataChristian P. WhitmanMircea PodarChristian SchlöttererMiriam Ragle AureChristina ErnstMiriam Szurman-zubrzyckaChristine BojanowskiMirjam SpengelerChristine CoustauMirjam Spengeler-FrischknechtChristine KottaridiMirko ManchiaChristine SinoquetMirosław TyrkaChristophe EscudéMiryam ValenzuelaChristophe HitteMitsuhiro MoritaChristophe PraudMitsuru NakazawaChristophe TrehinMitsuru SakaizumiChristopher CarrMiyuki MekuchiChristopher J. BrandlMohamed Abdel-NasserChristopher SauvageMohamed El-EsawiChristos DelidakisMohamed Raafat El-GewelyChristos RumbosMonica CappettaChunghee ChoMonika ChudeckaCiarán KellyMonika Stefaniuk-SzmukierCiro Rivera CasasMoosa MohammadiClaire FrancastelMuhammad Amjad NawazClaire WadeMuhammad HeidariClaire-Marie DhaenensMuhammad Jasim UddinClaudia BustamanteMukhtar AhmedClaudia KleinMunira BasraiClaudine MontgelardMurali VijayanClaudio AfonsoMurali YandaCláudio De OliveiraMuthu ThiruvengadamClaus WilkeMyunggi YiClément GilbertNabil EidClément GoubertNadine HannaClement LagrueNagarajan RajuClévia RossetNaim KhanClinton C. MacDonaldNakamichi NorihitoClizia VillanoNam V HoangColleen CronigerNaoaki SakamotoCongcong YinNaoki KanomataConstantine GaragounisNaoko TakezakiCorey GilesNaotake KonnoCorneliu TanaseNaoyuki KataokaCothran Ernest GusNaresh Doni JayaveluCraig HodgesNatália MarquesCraig MillerNatalie ClarkCristian P. MoiolaNatalie J. ForsdickCristian SmerdouNatascia TisoCristina AzevedoNatasha A HamiltonCristina BombieriNatasha E. SchoelerCristina CozziNatasha OlbyCristina F. MarcoNathalia Fernandes FagundesCristina OviloNathalie BoudetCristina RomeiNathan B. SutterCristóbal Almendros RomeroNathan LanningCristopher HolleyNatsuko MiuraCsaba BödörNavarro EstanisCynthia BottemaNazli McDonnellCynthia M. BeallNazmul HudaD.S. VerbeekNedenia Bonvino StafuzzaDae Kwan KoNehme El-HachemDaehee HwangNeil GrahamDaekyu SunNeil JonesDamian GawelNélida Inés NogueraDamiano MartignagoNicholas B. LarsonDamu TangNicholas DelihasDan T. A. EisenbergNicholas J. BrayDana Maria CopoloviciNick BrewinDanelle K. SeymourNick CollinsDaniel A. KluepfelNick DervisisDaniel H. De La IglesiaNicola ChiarelliDaniel H. ShainNicolas JonckheereDaniel HusonNicoletta PedemonteDaniel LawsonNieves CapoteDaniel MacqueenNigel BurrowsDaniel MeltersNigel G. HalfordDaniel ParkNik KovinichDaniel PetersonNiki VassilakiDaniel RenčiukNikita KuznetsovDaniel RuncieNina BrutchDaniel TaillandierNiraj ShahDaniel WeinreichNitin K SinghDaniel WoodsNitin RajDaniela DamianiNitish MishraDaniela GradiaNobuyuki MizunoDaniela HoltgräweNoha MesbahDaniela Matias De Carvalho BittencourtNowlan H. FreeseDaniela MoralliNuruddin UnchwaniwalaDanielle S. StolzenbergNutricati ElianaDanon Clemes CardosoOana Alina ZeleznikDanuta I. Kosik-BogackaOgnjen PerišićDardiotis EfthimiosOgnjen SekulovicDaria A. AndreyushkovaOk-Su KimDario PalmieriOlaf NielsenDarren J. WightOlaia Martínez IglesiasDavid A. StevensonOle SkovgaardDavid ArnostiOleg GusevDavid BuckleyOlga A. KofanovaDavid ChakravortyOlga DolgovaDavid D. DuvernellOlga ScholtenDavid GilbertOliver K. GlassDavid HaakOlivier GoureauDavid HesslOluwatosin OluwadareDavid HiggsOphélie LebrasseurDavid HumphreysOriol GuitartDavid J. MartinoOsamu KawaseDavid LloydOscar Junhong LuoDavid M. IrwinOskar MusidlakDavid PhalenOswaldo Valdes-LopezDavid Sanchez-InfantesOttmar DistlDavid ShoreOxana KlementievaDavid W. TaylorPablo FonsecaDavid WelchPablo LibradoDavid ZappullaPAJ LeegwaterDawid WalerychPaloma MoránDawn H NagelPanos G. KalatzisDe ChengPanos V. PetrakisDebasis ChattopadhyayPaola NavarreteDeborah J MarshPaola VittoriosoDeborah J. GoodPaola ZuluagaDeepmala SehgalPaolo FontanaDeepti Nigam SinghPaolo FranchiniDeirdre DonnellyPaolo PelliccioniDeliang PengPaolo RibecaDemetra AndreouPaolo ZambonelliDenis J HeadonParameswari PaulDennis MillerPascal EscherDeqiang SunPascale QuignonDesiree WandersPasi K. KorhonenDesmond RamirezPasquale TripodiDeyou QiuPatrice BellotDiana BoraschiPatrícia D. FreitasDiana CoralloPatricia SchulteDiana CousminerPatrick O’DonoghueDiana ValverdePatrick OwiliDick RoelofsPatrick SinnDidier PeltierPatrick SipsDiego GaleanoPaul SpellmanDiego García-AyusoPaula FernandezDieter P. ReinhardtPaula Maria MoreiraDietrich A. RuessPaulina García EusebiDietrich GotzekPavan Kumar PuvvulaDimitar VassilevPavel AnzenbacherDivya ChandranPavlína MáchováDmitrij FrishmanPaweł KonieczkaDmitry O ZharkovPaweł MackiewiczDoil ChoiPedro A SansberroDolores Gareth R. EvansPedro AlmeidaDolors VillegasPedro DoradoDominik BeckPedro SerralheiroDonald A. HunterPei-Hui WangDonald J ColganPeipei WangDonato MalerbaPeixin WuDongsup KimPeng JiDongya YangPeng YuDoris LucyshynPeng ZhangDr. Edoardo MalfattiPengda LiuDusana MajeraPeng-Hui WangDu-Xian TanPeppi KoivunenE.H. HoefslootPeter BulliEd SmithPeter CrispEduardo Andrés-LeónPeter EllisEduardo Mateo-BonmatíPeter FieldsEdward A. Ruiz-NarváezPeter GronesEdward K. GildingPeter HoudeEdward L. BraunPeter KaňuchEdwin WangPeter LyEdwina McGlinnPeter M. GresshoffEG BouldingPeter ScottEgor B. ProkhortchoukPeter SudmantEike StaubPeter WehlingEileen JaffePetrus TangEileen M. Roy-zokanPhilip CarellaEirini KanataPhilip ZegermanEisei SoharaPhilipp H. SchifferEizo TakashimaPhilippe BarreEl˝od Ern˝o NagyPhillip SponenbergElaine TaylorPia GallanoElaine TuomanenPierpaolo TrimboliElena De FelicePierre GrèveElena FeraruPierre-Antoine DefossezElena RaimondiPierre-François RouxElena Shekhovapierre-olivier angrandElena SticchiPiyush BaindaraEleonora LeucciPranav SahuElias KaiserPrashant MisraElide FormentinPrashanth AnamthathmakulaEliezer E. GoldschmidtPriya KatyalElisa PorcelliniPriyanka MishraElisabeth JonasProf. Dr. Kandasamy SaravanakumarElisabeth LabruyerePui Ying LamÉlisabeth Le Bihan-DuvalPushpinder BainsElisabeth MangoldPutty-Reddy SudhirElisabetta TabolacciQin LiElise PelzerQingde WangElizabeth A. HellerQingxiang ZhouElizabeth DuncanQingyu WuElke M. Van VeenQiong ZhangElsa SverrisdóttirQuan ZouElvira Sahuquillo BalbuenaQuanhu ShengElzbieta PluciennikQuanxi LiEmiley Eloe-FadroshQuentin CronkEmilia BagnickaR. Alexander PyronEmilia HadziyannisR. Keith SlotkinEmilie BrassetRachael WalkerEmilio CusanelliRachel E. KerwinEmily ClarkRachel J. O’NeillEmine Zeynep Erson-OmayRachel MertzEmma ThomsonRachita YadavEmmanouil MarkakisRadbod DarabiEmmanuelle BourneufRadhey S. GuptaEmmanuelle FabreRadhia M’kacherEmmanuelle LeratRadim BlažekEmrin HorgusluogluRadovan KasardaEnrique LemusRagupathi NagarajanEnzo MarteganiRahman Rahman PourEran ElhaikRahul ChandnaniEran MukamelRahul DuttaEran TauberRakesh UpadhyayErhard BremerRalf EinspanierEric CalvoRalph G MeyerEric JakobssonRamesh BalusuEric O’NeillRamesh BhatEric RuellandRamesh KatamEric T. StafneRamesh KumarEric TothRamesh VetukuriErica SalvatiRamsey LewisErica V. ToddRanjit Prasad BahadurErik ErsmarkRaphael MercierErik GarrisonRaphaela GeorgErin M. BrannickRaquel ChavesErin M. RehrigRaquel EchavarriaErinija PranckevicieneRaquel L. ChanErnesto Soto-ReyesRaquel Martin-FolgarEROS Lazzerini DenchiRaquibul HasanErtugrul FilizRasa LiutkevicieneEsteban Orenes PiñeroRasika HudlikarEswar ShankarRatan ChopraEtienne ChalletRatnadeep MukherjeeEtika GoyalRazmik MirzayansEttore MoscaRebecca D. DoddEugenia SanchezRebecca GladstoneEva TrevissonRebecca GraffEva VarallyayRebecca PoulosEvandro Marsola MoraesRebecca TallmadgeEvangelia AvramidouRebecca TarvinEvangelia MorouReema SinghEvangelos AxiotisRemco StamEvelina Y. BasenkoRémy MerretEvert M. van SchothorstRenata FreitasEvgeniy S. BalakirevRenata Jaskula-SztulEwa JablonskaRenata Toczylowska-MaminskaEwa NiewiadomskaRené Massimiliano MarsanoEyal SeroussiRenee DaleEzequiel TolosaRenée Laufer AmorimFabian FriesRenesh BedreFabio A. MarchiRenier Velez-CruzFabio CorredduRenjie TangFabio ManfrediniRenu PandeyFabrizio LombardoReyes BenllochFadi NabhanRicard AlbalatFady Hannah-ShmouniRicardo BenaventeFan LinRicardo De Matos SimoesFan ZhangRiccardo Aiese-CiglianoFang Rong HsuRiccardo PecoriFang ZhuRichard BurkeFang-xiang WuRichard M. BostockFanwang MengRichard R.-C WangFatma AydinogluRichard ThompsonFay BetsouRick RussellFederico ArielRie Shimizu-InatsugiFederico SebastianiRigmor C. BaraasFei HeRita TeixeiraFeixiong ChengRita UlloaFelix Jimenez-RondanRob W.J. CollinFélix MachínRobert A GrahnFelix VauxRobert DebuchyFeng CuiRobert FarrellFengjie SunRobert J. DuronioFernando Puente-SanchezRobert J. MorellFernando RendoRobert N. EisenmanFidel Ovidio CastroRobert RutkowskiFilipe PereiraRobert SchlabergFinn Skou PedersenRobert Van LooFiorentina AscenzioniRobert Van WaardenburgFlor De Fátima Rosas CárdenasRoberta BergeroFlorian LeeseRoberta Di RosaFlorin George HorhatRoberto Zenteno CuevasForbes MansonRobin WaplesFranca PellicciaRoderick C. SliekerFrances EdwardsRodney ScottFrancesc PiferrerRoel SchaaperFrancesc Xavier AvilesRoger LengFrancesca M. PisaniRohit GundamarajuFrancesca FerraraRohit KoloraFrancesca SparvoliRok TkavcFrancesco GentileRoland G. HuberFrancesco MusacchiaRolf W. StottmannFrancesco NeriRoman HobzaFrancesco Saverio SorrentinoRomonia ReamsFrancis B. NtumngiaRonald F. TurcoFrancis FangRonald LubetFrancis Morais Franco NunesRonald PeckFrancis NanoRoopa ThaparFrancisco J. EnguitaRosario GilFrancisco MirallesRosella EliseiFrancisco Rodriguez TrelleRoser Gonzàlez-DuarteFranck DumetzRudolph LeibelFrancois Pacquet-DurandRui Monteiro De Barros MacielFrank CoopmanRui OliveiraFrank DeanRuijuan LiFrantišek MarecRuilong ShengFraser ClarkRupert HocheggerFrauke CoppietersRussell SchwartzFred DambergerRuth ChadwickFrédéric BrunetRyan B. MacDonaldFreya MowatRyoko Machida-HiranoFriedhelm PfeifferRyszard BraczkowskiFuhai LiSabyasachi DasFulvio Della RagioneSacco C De VriesFulya TaylanSachin TeotiaFuyou FuSae Woong ParkGabriel CastrilloSaed KhawaldehGabriel MarkovSafa CharfeddineGabriel RodriguesSahra UygunGaëlle Le GoffSai ZhangGang LiSaivageethi NuthikattuGang LiuSakharam WaghmareGang YuSalim Timo IslamGangning LiangSalvador MireteGarth SanewskiSam MannaGary S. JohnsonSamy Y. LamouilleGary StackSanaa EissaGayathri RamaswamySandeep MarlaGayatri MittalSandra StudenikGemma MarfanySangkyu KimGeneviève Héry-ArnaudSang-Wook ParkGen-Hung ChenSanna OlssonGeoff NichollsSantanu BiswasGeoff ShawSara BergonziGeorge C DiCenzoSara MarchianiGeorges VassauxSara RicardoGeorgina StebbingsSara ZaccaraGeorgios D. KoutsovoulosSathya PuthanveettilGeorgios LiakopoulosSatya KotaGeraint ParrySavvas GeorgiadesGérard GuédonSayaka MiuraGerard MuntanéScott KaufmanGerardo García-Díaz BarrigaScott LeiserGerben ZylstraScott YoungerGermán MartínezSebastian PitaGerolamo LanfranchiSebnem Ece EksiGetinet Mekuriaw TarekegnSeema MahmmodGhyslaine Bruna Djeunang DonghoSeiichiroh OhsakoGiacomo De PiccoliSeiji TanakaGiancarlo PoianaSeisuke KimuraGianni TacconiSeonhee KimGianpiero MarconiSerap YalçınGiedrius GasiūnasSerena SanulliGil AtzmonSergio GiannattasioGiorgio MalpeliSergio MinucciGiovanna SerinoSergio Santander-JiménezGiovanni ChillemiSeung-Chul KimGiovanni NieroSeyun KimGiuseppe MatulloShabir Hussain WaniGiuseppe PugliaShahan DerkarabetianGiusi ZainaShahid SiddiqueGkikas MagiorkinisShailendra MauryaGlenda SobeyShalini OberdoerfferGlenn GerhardShalu JhanwarGoda VaitkevičienėShang MaGonzález Vasconcellos IriaShaolei TengGoran ZdunićSharmila GhoshGötz HenselSharon M BrookesGracjan MichlewskiShawn ThatcherGraham A. McCullochShereen SabetGregorio SerraShibiao WanGregory CookShihao SuGregory LavieuShih-Hsing LeirGregory PoonShihui JiaoGregory YochumShilpa SampathiGuijie ChenShimna SudheeshGuillaume DevaillyShingo KikutaGuillaume PilotShingo SakamotoGuillermo De CárcerShin-ichi YokotaGuillermo GiovambattistaShin-ichiro TakebayashiGünter LochnitShinsaku ItoGurvinder KaurShipra GroverH. Hamdan FedaShireen LamandeHai WangShota NishitaniHaibo LiuShrestha Sinha RayHaichuan WangShuen-Ei ChenHala AbdelmigidShuhei SegamiHaley WyattShuifang ZhuHamed BostanShuji OginoHamid BeikiShujian YuHannenhalli SridharSibum SungHans-Wilhelm NutzmannSiddharth SridharHarley SmithSigal Ben-ZakenHarold CorkeSigmar StrickerHarry BrumerSilvana PopescuHaruhiko FujiwaraSilvia Anna CiafrèHarumi JyonouchiSimone T. SredniHasna NjahSimonetta FrisoHeath BlackmonSimran MaggoHeather ShiveSirkka Kyostio-MooreHeba EbeedSmita KulkarniHéctor CandelaSofia OrtegaHector EscrivaSofie SymoensHee Bok ParkSong Beng KahHee-Jong KohSong-Tao LiuHee-Sung ParkSónia C. Da Silva AndradeHeeyoun BunchSonia GarciaHeike H. ZimmermannSophie De VriesHélder SpínolaSophie J.M. PiquerezHelen PiontkivskaSophie LeranHelen TempestSophie ShawHelena TrindadeSoumyaroop BhattacharyaHelene Robert BoisivonSridevi SureshkumarHélène SanfaçonSridhar JarugulaHelmuth HaslacherSriharsa PradhanHemant KhannaSrinivas SomarowthuHenk FranssenStacey SmithHenrique FerreiraStefan DennenmoserHenry MüllerStefan JouannicHerman E. PopeijusStefan ProstHerminia De La VargaStefania NobiliHernan Diego FolcoStefanie KellnerHervé SeligmannStefanie LehnerHideaki Mabashi-AsazumaStefano CagninHieronim JakubowskiStefano RomanoHimabindu GaliSten AnslanHindra HindraStephane RetyHiraku OnoStephanie Lovinsky-DesirHirofumi NakaokaStephanie MarciniakHiromi Kajiya-KanegaeStephen D BarrHiromichi SakaiStephen HowellHironori KawakamiStephen J BushHiroshi FukushimaStephen J. LalorHiroshi MiyamotoStephen KearseyHiroshi SatoStephen L HartHiroshi SugiyamaStephen SpatzHirotaka MiyamotoSteve DuncanHirotaka TomiyasuSteven FreedbergHiroyuki OkunoSteven J. KlostermanHollie PutnamSteven L. ParkerHong AnSteven LevyHong JiSubham DasguptaHonghong WuSubir SarkerHosam O. ElansarySudha SharmaHossain FirozSue Jinks-RobertsonHossam El-Sheikh AliSugandha SaxenaHouria DaimiSulay TovarHoyeun KimSumanta BasuHua ZhouSumit AgarwalHuan QiuSun HuhHuanmin ZhangSung-Ryul KimHuanyu WangSungwon JungHubertus Van HensbergenSunil KumarHugh Douglas GooldSunitha SukumaranHugo Fernández-BellonSupratim BasuHui ChenSuraj KapaHui-hua ChangSuresh DamodaranHung-Yi ChuangSurong ShuaiHuzefa RajaSusan BirketHyeon-Su RoSusana DunnerHyun Ji NohSusana FernandesHyun ParkSusana LozanoHyungdon YunSusana Olmedillas LópezHyunjae R KimSusanne ErikssonHyunju LeeSusanne SchillingHyunju RoSushama SivakumarIan HicksonSuzanne DawidIbrahim ElbasyoniSvetlana CherlinIchiro HirataniSvetlana DeryushevaIchiro MatsumuraSwapna Priya RajarapuIchiro NakagawaSylvie HermouetIda OreficeSylwia JafraIgnacio Del CastilloTaina CardosoIgnacio EzquerTaiping ChenIgnacio FernándezTakahiro NakayamaIgnacio MarínTakahito SutoIgor E. DeyevTakashi KuramotoIkuo MiuraTakashi TokinoIlaria PelusoTakashi YoshidomeIlia FishbeinTakehisa IsemuraIl-Keun KongTakuma IshizakiIndrajit MajumdarTakuro NakagawaIngeborg Johanna Maria DhoogeTamara HendricksonIngram IACCARINOTaniya MitraInmaculada Martín-BurrielTarah LynchInna KuznetsovaTarun SharmaIoakim SpyridopoulosTasuku ItoIoanna AntoniadiTatiana TatarinovaIoannis GanopoulousTatjana KleinowIrena RotermanTatsuo KidoIrene StefaniniTaylor WeissIreneusz LitwinTentsao WongIrina Goldenkova-PavlovaTeofil NakovIrina HashmiTereza ŠevčíkováIrina MakarevitchTerje RaudseppIsabel Anna Maria GrohTerri BeatyIsabel MafraTetsuya KoideIsabel MarquesTheetha PavankumarIsabella FincoTheodora FarmakiIsabelle GaillardTheodore LaetschIsabelle HenryTheresa M. CaseyIsaiah TaylorThomas A. CiullaIsidoro FelicielloThomas ArendtItamar SimonThomas BlankersIva BoušováThomas CarrollIvan MinkovThomas CaspariIvan MolinerisThomas Dugé De BernonvilleIvan SimkoThomas FriedmanIvan Y. IourovThomas G. MinehanIvana ViolaThomas HartwigIwao KukimotoThomas J. BachJ. David WarrenThomas M.A. ShafeeJack D. SunterThomas PreissJaco GreeffThomas SwerczekJaco VangronsveldThomas TietzeJacob MuellerThomas. PauschJacqueline SmithThorsten AllersJaebum KimTiago FernandesJaeyoung ChoiTianhua NiuJagdeep WaliaTibor CsorbaJaime Barros-RiosTihomir KovačJaime RomeroTilottama BiswasJaime-Amadeo BustosTim BeissertJAIPALREDDY PANGATim GearyJaishri O. BlakeleyTim Van DammeJakob FrankeTimo EngelsdorfJakub SkorupskiTimothy C. CoxJames AlbertTimothy DoranJames B. JaynesTimothy JamesJames Baldwin-BrownTimothy John MahonyJames D. JohnsonTineke L LenstraJames D. SutherlandTiziana SgammaJames DeCaprioTobias GorisJames ForneyTodd P. MichaelJames J DriscollTodd R. SteckJames M. ConnortonTomasz CzechowskiJames P. BennettTomasz HuraJames R. P., WorthTommaso PippucciJames WilliamsonTomoki KoshoJamie L. KostyunTomoyoshi KomiyamaJamie O’RourkeTorsten KrudeJan BenadaTorsten OchsenreiterJan BrosensTorsten ThalheimJan De RiekToyota KenjiJan LaRocqueTrine BildeJan SchirawskiTsering StobdanJana MoravcikovaTsukasa FukunagaJana PetrkovaTsunehiro AkiJane Geisler-LeeTsung-Hsien LeeJanina PetkevičienėTsung-I HsuJanosch HellerTulika SrivastavaJared NordmanTuri KingJaroslav JelinekUgo AlaJarosław PaluszczakUllrich KristianJason StewartUlrich DesselbergerJavier BrumosUlrich KückJavier Gallego-BartoloméUlrich PfefferJavier Ruiz-EderraUmakant SharmaJavier SantanderUmesh ReddyJean Michel GibertUn-Kyung KimJean-Baptiste BouléUrs RueggJean-Emmanuel BibaultUrszula OleksiewiczJean-Heinrich DaugroisVaiva LesauskaiteJean-Lou JustineVajiheh Safavi-RiziJean-Marc EscudierVal C. SheffieldJean-Marie ExbrayatValentina IotchkovaJean-Pierre RoussarieValentina LiakinaJeff KiddValentina MassaJeff StuartValentina NardiJeffery AdelbergValer GoteaJeffrey CraigValeria NaimJeffrey KimbrelValeria PrigioneJeffrey SchoenebeckValerie ChewJehannine AustinValerie WardJemma SeyfangVanessa Meier-StephensonJennifer CoughlanVânia Marilande CeccattoJennifer MorrowVasco ElbrechtJennifer SonesVasiliki KalatzisJens TetensVasu KommireddyJeremy AustinVéronique BergougnouxJeremy S. EdwardsVibha SrivastavaJesse J. SwenVictor AsensiJessica PetersenVictor FaundezJessica S. WilliamsVictor G. LevitskyJesús Gómez-zuritaVíctor García-GaytánJesus Maria Hernandez RivasVictor Ruiz-ValdiviesoJia-Ming ChangVíctor Sánchez-MargaletJian ZengVignesh DhandapaniJiang ShuVijay GahlautJiann-Chu ChenVijayalaxmi GuptaJiaxu LiVijayaprakash SuppiahJia-Ying ZhuVincent CousthamJie SunVincent KellyJikui SongVincent P. DiegoJim HuVincenzo LionettiJim PetitteVincenzo ParrinoJin ZhangVirgilio SacchiniJindrich CitekVirginie BourionJingyi Jessica LiVishnu DileepJinling HuangVittoria RoncalliJinsei JungVivian Capilla-CapillaJiří HrdýVladimir LazarevićJiri KoreckyVladimir MegličJitendra KumarVladimir TeifJiyang CaiVladimir VladimirovJo HandelsmanVolkan SayinJoanna Achinger-KaweckaW. Steven WardJoanna Beata Jankowicz-CieslakW.S. DavidsonJoanna Nowacka-WoszukWade BerritinniJoanne LittlefairWalid ELFALLEHJoao RodriguesWalter RocchiaJoaquín J. SalasWankun XieJoaquín Rodríguez-LeónWannaporn IttiprasertJocelyn PlassaisWayne VersawJoe SongWei LiuJoel R. DrevetWei ZhangJoel SharbroughWei ZouJoerg BurgstallerWeihang ChaiJohann BauerWeihua HuangJohann HeiderWen-Juan MaJohn AbramyanWenndy HernandezJohn AtackWenqing YinJohn MaloneWilliam Jon MeadusJohn McPhersonWilliam F. BlakelyJohn NitissWilliam N. MwangiJohn R. RohdeWilliam RuzickaJohn WardWlodzimierz OpokaJon BaxterWojciech J JanisiewiczJonas PaulsenWojciech MakalowskiJonathan A. ColemanWolf B. FrommerJonathan ClarkeWolfgang SadeeJonathan R SandyWujin SunJonathan R SkinnerWu-Jun GaoJonathan TurnerXavier Grau-BovéJonathan VelottaXavier MesseguerJong Gug KimXianfeng WangJong KimXiang YuJonghyun YunXianran LiJong-Kuk NaXiao ChangJordi MorenoXiaodong GuJordi TamaritXiaofei WangJörg MännerXiaoming ZhengJorge Fung UcedaXiaowei ZhanJorge Lozano-JusteXiaowen ShiJoris BeldXin BiJosé Antonio López-GuerreroXing ChenJose Barba-MontoyaXuan LiJose DieXuan XuJose E. Perez-OrtinXuehui LiJose Gustavo Ramirez-ParedesXuekui ZhangJosé Lima-BritoXuguang XiJose Louis Vega-PlaXun ChenJosé Luis García-MarínXutong WangJosé M Franco-ZorrillaYaacov Ben-DavidJose Manuel Borrero De AcunaYane-Shih WangJose Manuel Jose ManuelYang Jae KangJosefa AntónYang-Kao WangJosefa GonzalezYasuhiko KatoJosefin StillerYa-Xiong TaoJoseph Christie-OlezaYehoram LeshemJoseph D. PuglisiY-h. TaguchiJoseph F. WalkerYi ZhangJoseph HeitmanYibo DongJoseph YeelesYifei XuJuan C. SantosYing WangJuan Carlos García-RYingfang ZhuJuan GambiniYingjie SunJuan M. GuayasaminYinglin XiaJuan NogalesYingqian ZhanJuan Rodriguez-FloresYi-Ting LinJuan Rodriguez-VitaYitzhak HadarJuan SubiranaYixiang ZhangJudith L. RoeYohei NanjoJudith MontagYoko SattaJudith Van HoutenYong YuJudy BrusslanYongfeng GuoJugpreet SinghYong-Ick KimJulia MetzgerYongjin ParkJulia ShrubovycYonglong WeiJulian M CatchenYongqin WangJulian Tonti-FilippiniYoonkang HurJulie A. Maupin-FurlowYordan S. YordanovJulie GrahamYoshihiro ToyaJulien RougeotYoshihisa HirakawaJulien TremblayYosuke NagataJu-Ming WangYu NieJun ChenYuan WangJun LiYuchen YangJun MurataYu-Chung ChiangJun WanYuhan HaoJun WangYuhong TangJung Eun ParkYuji NagataJunghyun NamkungYuka IwasakiJunsoo ParkYumeng WangJustin A. PaterYun KangJustin AshworthYun-Fai Chris LauJustin P. Blumenstielyunhao wangKa-Chun WongYunke WuKacper ŻukowskiYuriy OrlovKai TangYvette Nout-lomasKalliopi ZachouYvonne OligschlägerKamakoti BhatZachary A. ChevironKamaldeep VirdiZachary S. TaylorKang Hee KhoZeeshan NasimKaren A. MatherZhang (John) WeihuaKaren KlyczekZhenlin HanKaren SanguinetZhi ChaoKari EkenstedtZhicheng DouKarim M. Fawzy El-SayedZhifu SunKarin NorénZhihang ChenKarine RicaudZhijun DuanKarl SeydelZhikai LiangKarl-Heinrich EngesserZhiqiang WuKarolina FučíkováZhi-yong LiKasavajhala PrasadZhiyong ZhangKasper L. AndersenZhonghua LiuKatarzyna DudekZhu ZhuoKatarzyna M. LisowskaZhuo ZhangKatarzyna MarciniakZippora (Zippi) BrownsteinKatarzyna Ropka-MolikZorana GrubićKate TsaiZuanning YuanKatherine A FantauzzoZubair AhmedKatherine A. HarrissonZubair M. AhmedKatherine BrownZuzana StorchovaKathleen E. Grogan


